# Observing ideologies. A systems-theoretical approach

**DOI:** 10.3389/fsoc.2026.1872106

**Published:** 2026-06-24

**Authors:** Kristoffer Klement

**Affiliations:** Faculty of Sociology, Bielefeld University, Bielefeld, Germany

**Keywords:** constructivism, functional analysis, ideology, Niklas Luhmann, reflexivity, second-order observation, systems theory

## Abstract

This article presents a conceptual analysis of Luhmann's systems theory as a framework for ideology research. To clarify the distinction between ideology/non-ideology as a guideline for empirical analyses, it derives a systems-theoretical concept of ideology from Luhmann's works, as well as the sociological foundations for its empirical operationalization. It does so by addressing two questions: First, it asks what we observe when we observe ideologies; and second, how we observe ideologies when we observe them. Both questions converge in an analysis of the functions of ideological meaning structures in systems operations with an understanding of their construction through second-order observations. As a result, ideologies emerge as social constructions of objectively given structures and operations with instructive, expressive, and reflexive functions that are hardly defined by strict boundaries but are often controversial and sometimes appear where we would not presume them at first glance.

## 1 Introduction: ideology research with systems theory

The following analysis assumes that ideologies are facts of our social life. Contrary to the claim that they are ‘dead' and scientifically useless, it starts with the claim that ideologies influence societies and therefore merit scientific attention. However, to make such a claim plausible, it must be clarified how ideologies can be observed as social facts. The present paper explores an answer to this question by discussing the theoretical foundations for a sociological observation of ideologies. As a conceptual analysis, it pursues the clarification of the concept of ideology within a broader social-theoretical framework and, based on that clarification, seeks to derive signposts for empirical research. Thus, its topic is the *outline of a sociological theory of ideology*.

Of course, there is no shortage of theories of ideology and approaches to their empirical observation (for overviews, see, e.g., Geuss, 1981; Maynard, 2013, 2017). Many of them work with a focus on politics by emphasizing the role of values and worldviews in political discourses, their interactions with other social structures such as the economy, or their functions for social domination and subjectivization. The following remarks are not intended to brush aside this long-standing tradition but rather to propose a new approach based on one of the most elaborated social theories to date: Niklas Luhmann's systems theory. Luhmann is certainly not known as a theorist of ideology—but that is precisely what motivates me to demonstrate his body of work as an ingenious foundation for the analysis of ideologies.^1^ The reflections presented here, therefore, understand themselves as a contribution to a *systems-theoretical theory of ideology*, which, at the same time, demonstrates the expandability of Luhmann's systems theory.

To build on Luhmann's systems theory is to adopt certain premises that underscore its particular suitability for a theoretically instructed observation of ideologies. Two points are especially important for what follows:

*First*, in the epistemological aspects of his theory, Luhmann assumes that we observe reality by distinctions and, on that basis, constitute our conceptions of reality. *Different concepts, different facts*, as Luhmann (2008b, p. 270) states. Our observations of ideologies are thus to be understood as constructive observations—and our understandings of ideologies as observational constructions. Such observations can occur on at least two levels: the general distinction ideology/non-ideology (e.g., liberalism in contrast to artworks or money) and the specifying distinction ideology x/ideology y (e.g., liberalism in contrast to socialism). Clarifying these two levels of distinction to explain what exactly we observe when we observe ideologies is a central aim of the present inquiry. For this purpose, it must address several problems that arise with the observational construction of ideological phenomena: the fuzziness of ideologies and their realizations, the sociality of their construction, and the relationship between observations and objective correlates.

*Second*, Luhmann's theory provides us with a specific mode of observation: *functional analysis*. According to this method, social phenomena are examined regarding which problems they address and to what extent different solutions (functional equivalents) become comparable from the standpoint of a given problem. From a systems-theoretical perspective, ideologies are thus grasped as problem-processing structures defined by the forms of their problem-processing, for which alternative structures such as law, science, power, and others may exist.^2^

Both premises—the explication of ideology-related forms of observation and the systems-theoretical form of observation of functional analysis—jointly specify the topic and structure of the paper. Accordingly, the following proceeds by first making meaning structures identifiable as ideologies on the basis of constitutive functions before reflecting on the constitutive influence of observation on ideologies. Chapter 1 is devoted to the functional definition of ideology and will lay out what we observe when we observe ideologies from a systems-theoretical perspective. Chapter 2 will transfer this mode of observation to the operative realization of ideologies in social systems: the fact of ideological communication. Because this reveals problems in identifying ideologies, Chapter 3 returns to the question of how ideologies are observed in society and thereby constituted. The concluding discussion summarizes the results and discusses the role of sociology as a specific observational position within society in order to present concisely the systems-theoretical yield for ideology research.

## 2 Ideologies: a functionalist definition

In order to observe ideologies, we should first define what we mean by ideologies. Luhmann's systems theory offers us a functionalist concept for this task as it defines ideologies in terms of certain social problems to which ideologies respond. These problems fundamentally include the reduction of complexity and the handling of contingency in the domain of value-guided action, as well as related aspects of legitimation, social integration, and practical coordination.^3^ In his analyses of ideology, he identifies three basic functions that are fulfilled through the operationalization of specific elements of meaning: a *pragmatic-instrumental function*, a *symbolic-expressive function*, and a *reflexive function* (Luhmann, 1982, p. 97–98). Their interplay defines the systems-theoretical concept of ideology that will be used here.

By the *pragmatic-instrumental function*, Luhmann refers to the selective orientation of actions through values and programs. He assigns values the function of serving as general selection perspectives for purposes and other behavioral programs such as norms, from which operations can draw more specific instructions and justifications. In this sense, values such as freedom, life, or prosperity symbolize abstract preferences for certain states of the world that define the scope of legitimate purposes, from which appropriate actions in turn arise, while other states of the world, purposes, and actions are delegitimized and neutralized (Luhmann, 1982, p. 97, 106–112; Luhmann, 2005, p. 72–76). Ideological orientation is thus understood as arising from a three-tier structure of general values, specific programs, and concrete operations, where ideologies symbolize the instructive nexus of the selection and justification of these values, purposes, norms, and operations. As “functionally regulative orders” for values, purposes, and norms (Luhmann, WId, n.d, p. 19), they incorporate structural expectations of behavior that tell us what is good, right, justified, and wrong.

However, the ideological repertoire of meaning is not exhausted by values and programs. As Luhmann notes in passing, it also includes “world-views, interpretations of history, interpretations of facts, as well as confirmations and distributions of competencies and duties” (Luhmann, 1982, p. 110). Such *descriptive, explanatory, demonstrative, and delegating semantics* support the orientation of values and programs by providing additional media of communication, rationalizing them, reinforcing their credibility, and increasing their acceptability. Consequently, ideologies are not only realized through explicit appeals to values and the justification of goals but also in positive self-presentations, product advertising, thematic emphases, role understandings, descriptions of human nature, and accounts of current world events in order to communicate their preferences and enrich them with additional information. The semantics of love—communicated through lyric poetry, prose, film, and social media—convey, for example, guiding ideas of correct feeling and behavior that encourage marriage and thus serve the purpose of species reproduction, as Luhmann argues (Luhmann, 1986, p. 145–154). Ideological elements of meaning thus also include semantics in a broader sense that express a particular worldview with its own, sometimes distorted, truths and thereby influence experience and action.

Yet ideologies not only provide information about what is to be done or what is the case; they also articulate desires, interests, needs, emotions, and problems. Luhmann refers to this as their *symbolic-expressive function*, in which ideologies “secure the consensus of those who will have to wait with their own values. An ideology must provide them with the certainty that they, too, will get their turn.” (Luhmann, 1982, p. 98). Luhmann does not elaborate on this mechanism in detail, but his analysis of the semantics of love makes it plausible that the symbols and practices of ‘true' love—e.g., a poem, a first kiss, or a marriage proposal—not only shape our feelings but also express them and thus appear attractive as subjective resonance spaces. More abstractly, this can be understood as the correlation of a knowing, believing, valuing, or feeling with a particular ‘state of being,' for which the concept of ideology, in Luhmann's interpretation, has stood at least since Mannheim (Luhmann, 1980, p. 56). Ideological symbols thus function as resonant references for subjective positions such as belonging to a particular class and thereby increase their orientation capacity because they include and shape the emotions and lifeworlds of those who bear an ideology.^4^

This becomes particularly clear in their *reflexive function*, which Luhmann closely links to the expressive one. By the reflexivity of ideologies, Luhmann refers to an opportunistic evaluation of values that manifests in a situationally appropriate interpretation and relevance of values and amplifies their complexity and evolution (Luhmann, 1982, p. 97–99, 101, 109). His basic idea is that value orders (just as goals, norms, and semantics) are adjusted to changing action needs and value realizations—in a broader sense, to problem constellations of social systems—in order to designate and legitimize values, programs, and operations effectively, and that precisely this adjusting variation defines the function of ideologies (Luhmann, 1982, p. 98). Therefore, “ideologies are to be understood as symbolic structures that allow the formulation of values to become reflexive and thus increase the scope and the complexity of this aspect of the decision-making process” (Luhmann, 1982, p. 98).

Consequently, ideologies symbolize complexes of values that oscillate between dynamic environmental adaptation and fixed symbols, with the oscillation playing a crucial role. As Luhmann emphasizes, certain symbols are typically removed from the process of revaluation to manage the risk of unlimited complexity (Luhmann, 1982, p. 98). Thus, ideological evaluations of values do not necessarily involve a total dissolution of all elements of meaning for the sake of adaption. Instead, ‘core' or ‘guiding values,' ‘basic needs,' and ‘irrefutable truths' function as foundational guardrails for ideological reflection, providing the ideology with a relatively stable identity. This becomes even clearer against the backdrop that social systems interact with their environments in a selective manner and never merely harmonize with them, which implies that their ideological adaptation also involves resistance and dogmatism, insofar as these help to address (environmentally induced) problems. In actually existing socialism, for example, authorized bodies such as the socialist party propagated an interrelation of “theory and practice” intended to update the values and goals of socialism in accordance with the current conditions of society and its environment, while adhering to the ‘irrefutable truth' of Marxism (Luhmann, 2010, p. 290–291, 305–330). Accordingly, a “true” Marxist, as Luhmann pointedly concludes, was distinguished precisely by staying up-to-date rather than just clinging to principles (Luhmann, 2010, p. 313). He had to know his capitalist enemy, one could say. However, he still and always advocated overcoming the fundamental evil of capitalism, as revealed to him by Marxism. Thus, his ideological reflections combined change and stabilization for a persistent yet adaptive orientation.

With this functional outline, the systems-theoretical concept of ideology should be sufficiently sketched for what follows. In short, we understand ideologies as *compositions of values, programs, and other semantics that are constituted through instructive, expressive, and reflexive functions*. Accordingly, their analysis in a systems-theoretical framework foregrounds a specific functionalization of certain elements of meaning. As for the general distinction between ideology and non-ideology, this means that we observe complexes of structures and operations with instructive, expressive, and reflexive evaluative functions, as opposed to other structures and operations that fulfill different functions or the same functions differently. Law, for example, can also guide and justify action through its conditional programs—but it typically refrains from evaluating values. Such evaluation may rather occur in the field of ethics—understood as a reflexive theory of morality (Luhmann, 2008a,b)—but, due to scientific requirements such as terminologies and methods (e.g., logic, deontology, consequentialism), it does so in a narrower range and manner than ideologies do in political or media contexts.^5^ Thus, ideologies differ from other structures, processes, and social systems in terms of their specific functionality.

However, this does not mean that ideology, law, and ethics are entirely distinct spheres. Functional and structural connections between law and ideologies, for example, are possible, as Luhmann states (Luhmann, 1982, p. 109, 111, 114). They become visible in labels such as a ‘liberal' or ‘fascist' legal order. The same applies to fields such as education, arts, economics, and politics, which are often infused by ideologies such as liberalism, capitalism, or socialism. Consequently, the boundaries between ideological and non-ideological structures are usually blurred. Ideologies are not delimited from other meaning structures by a specific form of meaning, such as a code or a specific medium, nor do they occupy an incomparable position in their individual functions. Of course, values play a central role in their reflexive instructions.^6^ But neither values nor the functions of instruction, expression, and reflexivity alone are exclusive features of ideologies.

So, what makes ideologies ideologies? The solution, in my view, lies in their *compositional character*. It indicates that it is the inherent interrelation of functions and meaning structures that differentiates ideologies in general and in particular. Not single values, programs, or world descriptions define an ideology such as liberalism in contrast to law or socialism, but rather their connection in the context of value reflections, practical instructions, and symbolic expression. Liberalism, for instance, can be described as a specific ideology because it reflexively prefers a strongly individualized notion of freedom over collectivist conceptions, justifies programs such as individually reasonable infection-control measures or individualized mate choice, and provides an expressive medium for the interest in private enrichment in the freedom of unrestricted trade. In all of this, it is not the description of the COVID-19 pandemic or generic notions of love and freedom that specify liberalism—but rather the interpretations and combinations of these elements of meaning within a nexus of instructive, reflexive, and expressive functions.

It should thus be emphasized that ideologies are defined as distinct phenomena only through *the relation of various components*. Not one individual function or symbol defines them, but their connections. Put simply, the functional mixture of practical instruction, symbolic expression, and value reflection enables a general identification of operations and structures as ideologies against functional equivalents such as law, art, or ethics. At the same time, the functional linkage of meaning structures and operations opens access to the empirical diversity of ideologies. An ideology such as liberalism is, in this sense, a composition of specific elements of meaning from the standpoint of practical instruction, whose symbols provide subjective possibilities of expression for those being instructed and which, at the same time, opportunistically evaluates the symbols of instruction. For the analysis of ideology, it is thus crucial to relate and contextualize single phenomena such as purpose specifications, action justifications, love vows, or evaluations. This becomes clearly visible in the operative reality of ideologies.

## 3 Observing ideologies: ideological communication

So far, we have clarified by which criteria ideologies should be observed from a systems-theoretical perspective. They are complexes of elements of meaning with instructive, expressive, and reflexive functions. The more concrete observation of ideologies now turns to their realization in social life—i.e., their realization as meaning structures and operations in social systems. In what follows, I focus on their operationalization as ideological communication since, in accordance with Luhmann's general theory of social systems (Luhmann, 1984, p. 191–241), this defines the primary mode in which ideologies are realized in social systems.

Thus, we must clarify what constitutes *ideological* communication. Considering the criteria developed above, this initially seems easy: *Any communication can count as ideological whenever elements of meaning like values, purposes, norms, and semantics are actualized within a nexus of instructive, expressive, and reflexive functions*. The communicative operationalization of an ideology can thus consist, for example, in the justification of military operations with arguments such as liberation and security against the background of a perceived threat; or in media portrayals of lovers whose happy ending culminates in marriage and family; or in the reflexive upgrading of responsibility as a guiding value against overly individualized notions of freedom to justify infection-control measures (for the German debate on this, see, e.g., Dirksmeier, 2020; Nullmeier, 2020).^7^ In each case, communications of this kind can count as ideological because they fulfill instructive, expressive, or reflexive functions in a broader nexus of meaning structures that is constituted by precisely these functions.

However, what looks simple at first glance proves difficult upon closer inspection. This is because, in many cases, it remains unclear how to draw a line between ideological and non-ideological communication, and how to attribute individual acts of communication to a specific ideology. For instance, is marrying for love and starting a family an exclusively ‘bourgeois' action, or can it also be a socialist one? Are the German Basic Law and the American Declaration of Independence ideological texts or not just as much political, legal, or even artistic ones? And does only the *affirmative* synthesis of individual freedom and social responsibility represent a reflection of liberalism—or does criticism of individualized freedom also count, since it prompts liberals to rethink their positions? As already noted, the dividing lines between ideology and (non-)ideology are by no means unambiguous.

But what about significant, indispensable symbols of ideologies such as freedom for liberalism or love for ideologies of love? Of course, buzzwords and guiding values like these are important markers for attributing a communicative act to a specific ideology.^8^ Still, this is hardly sufficient because it fails to do justice to the focus on functionality as the defining feature of ideological communication and to the empirical diversity of ideological communication and their orders of meaning. In short, ideological communication is not limited to invoking values and human nature to justify goals; it can also be actualized in mass-media reporting (Jensen, 2018; Lylo, 2016, p. 18; White, 2006), in linguistic intonation and the selection of music (van Dijk, 2024, p. 6), or in prices (Klement, 2026b)—that is, in a diverse semiosis of its symbolic forms (Fairclough, 2006), which also implies subtle and indirect modes of communication. Thus, what matters is once again not the individual form, mode, symbol, or act but their overarching connections. Instead of considering communicative events in isolation, ideological communication must be identified along its relations to a broader nexus of events, symbols, and functions. In this sense, for example, the expression of feelings in a marriage proposal is ideological if it simultaneously correlates with a conception of love that is bound up with the norms of heterosexual marriage and the goal of procreation, such that the value, the norm, and the purpose confer a deeper meaning on the proposal and on our feelings. Or in other words: *It is the context, just as much as the individual characteristics, that gives the individual element its ideological quality*.

The significance of practical contexts is, of course, well-established in ideological theory. Yet it can easily be overlooked and reduced to “what bearers of ideology are *saying*, [instead of] what they appear to be *doing*—e.g., lobbying for support, trying to persuade, satirizing others, pleasing the interviewer, saying what they ‘ought' to, etc.,” as Maynard (2017, p. 313–315, cited 313) notes in his reflections on contextualism in ideological analysis. Consequently, the implicit aspects of ideological communication must be considered beyond mere self-presentations and explicit statements—a postulate that perfectly fits with Luhmann's methodological consideration (Luhmann, 2013a, p. 13–14) that the context of communication must be transformed into text in order to reintroduce what the systems themselves exclude from communication. Thus, obvious communicative data alone cannot suffice for a systems-theoretical analysis of ideology; rather, the functions, operations, and forms of meaning kept latent by the systems and their ideologies must also be uncovered. However, the systems-theoretical approach presented here differs from that of Maynard and others because it focuses on the intersubjective and transsubjective communication process for a context-sensitive analysis, rather than on the (hidden) intentions and attitudes of the subjects, as we will see below. Based on this, it allows for a genuine and nuanced understanding of the nature and relevance of contexts.

With Luhmann, a relevant context of ideological communication is to be understood in three dimensions of social meaning: factual, social, and temporal. Factual, it is other elements of meaning—such as individuality and freedom—combined within a nexus of instructive, expressive, and reflexive functions that turn a marriage proposal into a manifestation of liberalism or economic prices into an expression of an ideology of ecological sustainability. Temporally, by contrast, each communicative act takes place at a particular moment in a particular situation or epoch and is integrated in an operative process that takes time itself. Precisely this processualism of communication, however, points to the social context, which consists of other operations and potentially forms into a social system. Thus, a marriage proposal is to be understood as an act of a modern liberal ideology of love if it was preceded by highly individualized courtship and followed by the founding of a nuclear family, so that individual freedom and species reproduction are realized as driving values and purposes in a system of intimate interactions. Similarly, higher prices for sustainable products may count as an expression of a sustainability ideology if companies justify them with ecological risks and sustainability strategies, which means that they occur within an organizational system with a comprehensive ecological orientation.^9^ By contrast, both cases should be regarded as non-ideological if the proposal is made solely for tax advantages and prices are set only based on market data. In that sense, the factual, temporal, and social context of communication is a crucial key to its ideological character—and to the challenges of observing it.

Relationality and contextualization—in short: the *systemic* character of structures, functions, and operations—are thus key aspects of ideological qualities that can help explain difficulties in identifying ideological communication. In the factual dimension, this becomes clear in Luhmann's triadic concept of social communication. In his view, communication consists of three elements: utterance, information, and understanding (Luhmann, 1984, p. 193–201)—or, in other words, three distinct horizons for identifying ideological meaning and functionality. Above all, the difference between utterance and understanding is pivotal here, since it forces us to distinguish two different interpretive accesses to communicative information while at the same time considering the communication process as a unity of both. What is meant and which problem is addressed by an item of information is determined differently by different communicators. Therefore, it is not only the intention on the side of utterance but its further processing in different reception contexts (i.e., in systems) that decides what functional meaning a communication acquires. A critique intended as rejection, for example, can contribute to the development and consolidation of an ideology if its problematization is used to reinterpret existing values, norms, and understandings of the world (see, as a classic on capitalism, Boltanski and Chiapello, 2005, p. 27–35, 96–99, 514–516). Consequently, the negation of an ideology on the side of utterance is not sufficient to assign a communication exclusively to one specific ideology (e.g., an anti-capitalist one), because such negation can also count as a functional manifestation of the negated ideology if it becomes part of its reflexivity. Or put differently: Since utterance and understanding are differentiated, communication is a realm of unintended consequences, making the analysis of ideological communication a mess.

Of course, the crucial role of understanding and its contexts in the formation of ideological meanings has been repeatedly emphasized by authors such as Hall (1980, 1985) and van Dijk (1998, p. 204–205, 2013, p. 180–188). However, the systems-theoretical analysis focuses neither on understanding alone nor on subjective attitudes and intentions, but rather on the communication process as a whole, in which attitudes, intentions, utterances, understandings, informations, meanings, and functions must be both distinguished and related to one another. This does not preclude the analysis of ideological attitudes and intentions; yet it warns against equating them with the meanings and functions of ideological communication. This differentiation is even confirmed by social-psychological ideology research, which, according to Jost et al. (2009, p. 312–313), indicates that the ideological attitudes of subjects are differentiated according to thematic contexts and system references: While one may be liberal in social respects, one can be conservative in economic respects. However, if ideological attitudes vary depending on the context and thus depend on social differentiation, their informative value regarding the meaning and function of ideological communication is secondary to the contexts. For this reason, analysis should abstract from both the subjects and the individual acts of communication and instead consider contexts as social systems that influence utterances, understanding, and attitudes according to their functional specifications.

Against this backdrop, we can now see that the ideological character of communication is inherently complex and ambiguous, which is why it cannot be adequately defined on the basis of a single perspective or event. It results from the chaining of multiple acts of communication, with the reception side playing a crucial role as the information-processing context. Identifying ideological communication, therefore, requires a context-sensitive relating of differentiated events, in which the meaning, effect, and attribution of individual statements change with circumstances such as specific system references. This poses particular challenges in the highly differentiated, polycontextural society that modern society can be understood to be with Luhmann (2013a, p. 13, 46–47, 2013b, p. 183, 313, 337, 343), since communicative acts can combine diverse meanings and functions and thereby produce an operational coupling of various functional systems here (Luhmann, 2013b, p. 8, 16, 115). Accordingly, modern communication is to be analyzed as multifunctional and multireferential^10^, which makes it difficult to cleanly distinguish ideological from non-ideological and differently ideological communication. Where the discourses of liberalism, capitalism, socialism, or fascism begin and end is often even more indeterminate—functionally speaking—than the boundaries of their ideological ‘factual systems,' since a socialist critique of capitalism can be appropriated within pro-capitalist discourses to consolidate one's own ideology, and thus becomes part of that discourse as well. As mentioned above, the communicative boundaries between specific ideologies are as fluid and fuzzy as their general distinctions to legal, aesthetic, political, or economic communication, each of which can contain ideological communication, because in the same communicative act different functions and meaning contexts can be realized. For this reason, a political science analysis of a pandemic can imply evaluations of values (e.g., Mohammed, 2020 on neoliberal notions of freedom), making it a potential driving force for the ideological reflexivity of a political party, while an author like Rawls can be “both a philosopher and an ideologist because his texts can be subjected to totally diverse analyses and can carry various meanings for different types of reading (and for different disciplines),” as Freeden (1998, p. 45) aptly remarks. Since texts are “multi-layered,” Freeden (1998, p. 45) correctly states, “they are capable of bearing more than one significant set of messages.”

This confirms once again that a communicative act can be relevant to various systemic contexts such as functional systems (science, politics) or scholarly disciplines, which in turn shape the communicative act according to their information-processing schemes, situations, and histories. Ideological communication can coincide with political and philosophical communication at the same time—for example, by legitimizing the exercise of political power through a theory of justice—while being shaped by these specific forms of communication and their systemic context. Accordingly, the factual meaning nexus of an ideology, which as such is established through functional relations, is constituted by the social system in which it is functionally processed: In liberalism, for example, the balance between individual freedom and social responsibility shifts in response to the demands of political power or educational challenges. However, a pivotal aspect of such constitution through system processing is not only the immediate ideological utterance or its singular reception but also the observation of utterance and understanding by those who utter, those who understand, and third parties. Put shortly: *What matters is the interpretative observation of ideological communication within a social system*. The social construction of ideologies is thus central to their constitution and identification.

## 4 Observing observations: the social construction of ideologies

The preceding considerations should have clarified three points: First, ideologies exist as relations among structural and operative elements; second, these relations are constituted through operations such as utterances, understandings, and observations; and third, these constitutive operations are shaped by the respective systems to which they refer. We can therefore conclude that the ideologies of social systems are produced by the operative processes of these systems. They are not objectively given facts like stones or stars, but socially made facts that emerge autopoietically from the communication of the systems. Accordingly, ideologies such as liberalism, socialism, anarchism, or environmentalism are constructions of ideology-related communication, including self-descriptions and external descriptions that define their functions, contents, operations, and thus their identities and boundaries. This is why they often appear fuzzy, fluid, and marked by interpretive struggles.

Once again, such insights are by no means new. From Marx's assumption that class-based power relations structure a society's ideological field (Marx and Engels, 2010, p. 59–66) to the subsequent thesis that ideological signs can be interpreted from multiple perspectives and express social struggles (Volosinov, 1973; Heim, 1982) to theories of antagonistically structured discourses and productions of meaning (Laclau and Mouffe, 1985; Fairclough, 2006, 2010; Hall, 1980, 1985, 1997; van Dijk, 1998, 2013), the socially immanent construction of ideologies has long become common ground in ideology theory.^11^ Or, as Weber (1992, p. 13–14) already put it succinctly: The spirit of capitalism “must be gradually *put together* out of the individual parts which are *taken* from historical reality *to make it up*,” (Weber, 1992, p. 13, emphasis mine) whereby different understandings may arise depending on the interest of the analysis. The systems-theoretical perspective both confirms these traditions and enriches them by explaining the social construction from a basic principle of modern system formation, which it intertwines with system-related functional requirements: higher-order *observation of observations*, or *communication about communication*.^12^ Social systems, in this view, do not consist only of actions and communications but are also shaped by the problem-oriented thematization of these actions and communications. What exists socially is, put simply, what societies perceive and tell about themselves in order to respond to their challenges.

The same can be assumed for ideologies. What turns hammer and sickle into a symbol of communism and a marriage proposal into a manifestation of a liberal script of love is our meaning-giving observation of these symbols against the background of problems such as the political unification of workers, fostering personal relationships, and starting a family. And what further elevates them to *iconic* symbols of communism or liberalism is their repeated presentation and thematization as ideological symbols against the background of the battle between competing configurations of society. As social facts, ideologies are thus substantially produced through second-order observation, i.e., communication about communication. They not only have a reflexive function by which they determine what values are (currently) relevant but are themselves products of reflection insofar as they are shaped through thematizations of ideological communication. As a consequence, what counts as liberalism, socialism, anarchist love, or a fascist family must be considered as a result of society's communication about liberalism, socialism, anarchist love, and the fascist family.

Since the forms and functions of communication influence the ‘material' content of ideologies in this process of second-order construction, diverse perspectives on and of ideologies emerge in differentiated societies. The economic, political, educational, or scientific understandings of liberalism, for example, most likely differ because values such as freedom acquire different denotations and connotations correlated with their functionalization in the respective systems. Liberalism in the political sense, for example, emphasizes freedom of speech and free elections, whereas economic liberalism highlights the freedom of private property and deregulation, while the scientific perspective sees the freedom of research and teaching as well as a research object. Concise what holds for ideologies is therefore what Luhmann (2000, p. 321) states for social systems in general: In the context of observed self-descriptions, ideologies *are* the same thing in different ways—and thus the unity of a multiplicity of observation modes.

In systems with various sources of self- and external descriptions, a polemogenic pluralization of ideologies thus seems hardly avoidable. The multiperspectival character of modern society makes any unity—and therefore any unambiguous identification—of ideologies a paradoxical, almost impossible, undertaking that can likely be remedied only through selective highlighting, privileging, ignoring, marginalizing, and suppressing certain descriptions of ideologies. Consequently, ideological conflicts are the counterpart to the differentiation of social systems, each of which defines ideologies based on its own observations and problems.^13^ This does not rule out cross-system isomorphisms and interconnections of ideological contents, nor the society-wide impact of particular ideologies such as liberalism or socialism. The various interpretations of liberal freedom converge, for example, in the absence of external interventions, also known as negative freedom. Most of the time, however, such context-transcending elements turn into abstract empty formulas and buzzwords whose concrete meaning can only be reconstructed in system-specific terms (see, on sustainability, Klement, 2023). In Luhmann's terms, the identities of ideologies are thus only graspable through shifting description contexts, whereas the flight into an abstract context-freedom would forgo what makes meaning identifiable in the first place: reusability across different contexts (Luhmann, 2000, p. 321, esp. fn. 4).

How this system-relative constitution of ideologies occurs in detail cannot be pursued further here. However, the present paper already provides its own example as it constructs and communicates a system-relative mode of observing ideological communication by presenting a scientific framework for the study of ideology. The social system of science within which it seeks to integrate determines its specific mode of observation and communication, as evidenced by its terminology, references to theories and empirical examples, and its line of reasoning, while the system-specific problem of producing truths about ideologies specifies its understanding of ideologies as social facts that must be accurately described. In this respect, the current considerations serve as a live example of the social construction of ideologies.

Almost incidentally, three important aspects can be drawn from this example that should be considered to understand the construction of ideologies properly:

*First*, ideologies are not fictions even if they are constructed through observations. Observing them rests on objective correlates—i.e., facts and data such as attitudes, symbols, expectations, and operations—that can be ascertained in social systems (despite all hermeneutic fuzziness). They are thus objectively given *and* socially made, which means, *how* they appear depends on the forms by which they are observed (e.g., as functional compositions of values, programs, and semantics); but *that* they can be observed at all is grounded in events, structures, and processes that are not arbitrarily invented by observation.

This becomes clear in the *second* aspect, which states that ideologies do not become part of social reality only through reflexive thematization but are already realized in pre-reflexive communication processes. For even if it is true that an ideology is identified by relating individual structures and operations through higher-order observations, the identity-forming elements of ideologies often emerge as “eigenvalues” of communicative reproduction (see on the concept of eigenvalues Luhmann, 1992b, p. 103, 2013a, p. 237). The repeatedly successful invocation of freedom, for example, has made it an almost indispensable value for every relevant ideology in modern Western societies, from liberalism to fascism—even if the freedom concepts employed sometimes degenerate into mere fig leaves for authoritarian desires (see, e.g., Brown, 2018; Amlinger and Nachtwey, 2022). Freedom is, in other words, a product of ideological reproduction. This does not diminish the relevance of communication about ideologies to the formation of ideologies, since the question of how freedom is functionalized in structures and operations that can be considered as liberal still points to a higher-order communication that subsumes these structures and operations under the label of liberalism. But it suggests that the identities of ideologies arise from a synthesis of the eigenvalues of ideological communication and the eigenvalues of communication about ideologies (such as liberalism or the concept of ideology). Or, put differently: Even though reflexive thematization makes ideologies recognizable as complex facts of the social world, it is nonetheless only *one* generative factor of the social world *among others*. It requires a point of reference that it cannot simply create out of nothing without claiming a divine position—that is, a position that brings forth the world and itself solely from its own imagination.

However, since no social observation can claim such a divine position without being regarded as eccentric, insane, or, at best, artistic, the social construction of ideologies cannot proceed arbitrarily. Instead, as a *third* aspect, it must be socially acceptable in order to be reproduced. Therefore, it must engage with the conditions for acceptance among its designated contexts (e.g., an audience or a specific voter group) in both an adaptive and formative way, including the acceptability-forming problems of these contexts. This is one reason why inclusions, exclusions, defamations, praises, alliances, and hostilities structure ideological discourses in differentiated societies: They shape the social acceptability of ideology-related communication and thus form the ordering patterns of social ideology production. More fundamentally, however, this reveals that any construction of ideologies proceeds from a specific context and thus can claim only context-relative validity—as well as context-relative particularities. To conclude the reflections on the scientific observation of ideologies, it is therefore advisable to discuss briefly some particularities of the observational context of the present paper.

## 5 Discussion: science in the battlefield

The aim of this text was to show that Niklas Luhmann's systems theory can provide a productive foundation for ideology research. Regarding this aim, the preceding considerations have demonstrated that his theory offers a functional concept of ideology that can be used both to analyze ideologies and to understand how the observation of ideologies plays a key role in their constitution. Put simply, ideologies appear as complex, autopoietically produced facts of the social world. They symbolize compositions of functionally specified expectations that are realized in nexuses of operations and, in their structural as well as operative interrelations, are shaped by context-guided self- and external descriptions. Thus, the essence of the concept of ideology proposed here lies in the argument that we understand ideologies as functionally constituted and socially constructed systems of structures and operations whose function is the adaptive integration of action-guiding and expressive symbols in relation to current social conditions.

A key implication of this perspective is that many seemingly isolated structures and processes can be viewed as elements of ideologies within various systemic contexts, so that ideologies, conversely, can be elements of various social systems. Consequently, ideologies may often seem somewhat vague, which certainly makes them more difficult to study empirically than if they were defined as clearly delineated value systems. However, this is precisely the point of the systems-theoretical approach: Ideologies are not clearly delineated objects. Rather, they are autopoietically constituted, fluid, and often blurred differences between compositions of operations, structures, and functions that may overlap with other social phenomena such as law, politics, ethics, art, etc. Accordingly, their observation as facts of the social world is not simply based on objective evidence but on a social construction through higher-order observations of ideological structures and operations. And therefore, their specific, various forms primarily rely on self-descriptions and external descriptions *of* ideologies *as* ideologies. Or in other words: What ideologies *are* depends on *how* they are observed and depicted.

This brings us, consequentially, to the question of what role the scientific system, as a specific observation context, plays in the social construction of ideologies. Luhmann offers instructive pointers here as well. In his farewell lecture titled “*Was ist der Fall?*” *und* “*Was steckt dahinter?*“ *Die zwei Soziologien und die Gesellschaftstheorie* (“What is the case?” and “What lies behind it?” The Two Sociologies and Social Theory) (Luhmann, 1993), he sketches three possible paths for sociology. In a classical sense, sociology seeks, on the one hand, to determine which social facts occur and what entities such as societies, institutions, and organizations are, while, on the other hand, it aims to show what lies behind these facts in terms of latent but relevant phenomena and processes. In this dual role, sociology functions as a descriptive, explanatory, sometimes unmasking self-observation of society. Since the forms of observation used in doing so, however, arise only from society itself and significantly constitute their object of observation, he moreover assigns sociology the task of observing the very distinctions by which society observes itself. Sociology then assumes the function of reflecting the repertoire of societal (and thus scientific) observation and thereby indicating other possibilities to observe. Its métier is not only to produce constative, explanatory, and enlightening statements but also to describe *how* facts and latencies are grasped. In this way, it can finally act as a potential driver of contingency in society's self-description.

All these paths can be transferred to the field of ideology research and are, to some extent, already present in the current paper. To the question of what is the case, sociological research responds with descriptions of ideological facts—for instance, by analyzing ideological communication functionally, as proposed here. In this way, it formulates its own voice within the orchestra of society's observations of ideologies—a voice that is neither simply superior to others nor merely equal to them. Because of the epistemic expectations that science, compared to other social systems, has made its profession, it can serve the social construction of ideology—just as in other domains of knowledge—as a special corrective. It does so by producing theories, concepts, methods, and analyses that subject society's ideological constructions to a plausibility check, discussing which attributions of function, meaning, and relations correspond to facts, including the constructive communication about ideologies themselves. In this sense, one might clarify, for example, whether the glorification of liberalism as an ideology of just freedom corresponds to real practices of justification and distribution that are labeled liberal or whether instead its denunciation as a veil for domination is correct. Although science cannot dictate the definition or substantive elaboration of ideologies, it surely can claim to have a voice of particular weight.

This becomes even more evident as it reveals what lies behind the obvious ideological phenomena. Classically, this figure has been addressed by authors such as Marx and Adorno as the exposure of society's false appearance and of subjects' false consciousness. Luhmann takes up this traditional function when he urges functional ideology analysis to uncover, alongside manifest values and purposes, also the latent consequences and functions of ideological selectivity in order to improve the complexity, evaluation, and variation of ideological orientations (Luhmann, 2005, p. 77). Complementing this, one might, in line with his sociology-of-knowledge research program—which saw in the concept of ideology a predecessor of its own intellectual history (Luhmann, 1980, p. 12–13, 56–57)—highlight correlations between ideologies and other social structures, especially functional differentiation, in order to better understand the selection and problem contexts behind ideologies for the analysis of functional equivalencies.

Finally, in the sense of observing society's forms of observation, sociological ideology research is called upon to describe the social construction of ideologies and to ask about its functions. Beyond describing ideologies as facts, it investigates how and why societies produce ideologies—that is, how ideology-related observations and attributions actually proceed. For this purpose, it analyzes relationalizations and boundary drawings, deconstructs schemes of distinctions, and questions references to objective correlates in order to reveal how ideologies are *made*. In doing so, it can expose the selective and contingent character of given ideologies, test them against epistemic, functional, and normative criteria, and encourage improvements. Processes of this kind are long established in the manifold discourses, critiques, and deconstructions of prevalent ideologies. Systems-theoretical sociology thus merely aims to equip what already exists with a self-consciousness about its own activity and to expand its set of instruments. Its contribution could involve explaining the social functions of distinguishing between ideologies and non-ideologies—such as the (de-)legitimization of political enemies—analyzing what has become socially relevant as liberalism, fascism, or a new ideological formation, and examining the extent to which ideological content, consequences, and functions have been overlooked thus far. As a result, systems-theoretical ideology research can significantly enrich society's ideology-related self-understanding and thus continue an important legacy of its critical enlightenment: sharpening awareness of what societies truly must deal with in terms of their ideologies.

This, of course, also puts the scientific understandings of ideology up for discussion, both in general and in particular. What the scientific treatment of ideologies says about the social reality of ideologies becomes a distinct field of research, traditionally shaped, for example, by the controversy between neutral and critical concepts of ideology, in which social (ab-)uses of ideologies are reflected. From a systems-theoretical perspective, one suggestion here is to overcome the schism between neutral and critical concepts through a critical analysis of functional equivalencies (see, regarding Luhmann, Klement, 2026a, p. 200–253) and to adapt other methods of ideology analysis in an equally constructivist and functionalist direction. As shown here, this would imply, in particular, emphasizing the factual, social, and temporal contexts of the functionalization of elements of meaning, including the hermeneutic observer as a context and as context-dependent.

All of this is based on the systems-theoretical premise that ideologies should be understood in functionalist rather than ontological terms, which means relating them to problems and comparing them with alternatives rather than exposing them as distorted beliefs. From a functionalist perspective, as Luhmann (2005, p. 69–75) explains, ideological thinking is characterized precisely by the fact that it is interchangeable in its functions of orientation and justification and emerged historically at the very moment when values, norms, and purposes were no longer regarded as truths of cosmological order, divine will, or human nature. Accordingly, the systems-theoretical analysis of ideology must focus on the social processing of radicalized contingency in the realm of social orientations—for which the social construction of ideologies serves as a paradigmatic example. Ideologies should not be reduced to supposedly essential meanings or eternal substances but rather be examined as social reference points for functional variations of orientation symbols.

From this perspective, the variety of ideological manifestations becomes recognizable as an *ideological* variety through the functions of instruction, expression, and reflection, which is further differentiated by the variety of ideological fulfillments of these functions in concrete systems, since ideologies connect different functions and have different functions in different systems (Luhmann, ZK II, 1961–1997, 3411/9,1). In other words, ideologies can neither be fixed to a specific operative substance, nor do they manifest only in particular values, goals, or expressive forms. Rather, they form compositions of variable meaning structures whose compositions, boundaries, and operationalizations are shaped by their system-relative multifunctionality. This is why a systems-theoretical approach calls for analyzing ideologies as socially established reference symbols for multifaceted orientation performances, to which their content and empirical manifestations are aligned, instead of only describing value systems, attitudes, or practices of justification.

In summary, Luhmann's systems theory thus favors a form of observation that could be called *constructivist functionalization*. From this perspective, what defines liberalism, socialism, or feminism depends on the functional requirements of social systems, which act as filters for identity-forming self- and external descriptions. This is, of course, itself just one mode of observation among others. It can be supplemented by other modes and must not limit itself to the factual dimension, as demonstrated here. Beyond the relations among values, programs, and semantics, sociological observation must direct its attention to the social structures and mechanisms of ideological construction processes. Organized production of ideology, interpretive monopolies and hegemonies, the emergence of communicative eigenvalues, and histories of ideological evolution are, in this sense, necessary ingredients of an ideology research program that knows how to locate ideologies within the autopoiesis of social systems. This last point finally formulates Luhmann's impulse for ideology research: to observe the creation of ideologies through social communication more precisely and, in doing so, influence this creation.

For this purpose, his theory of modern society finally offers an ingenious combination of communication theory, evolutionary theory, and differentiation theory—resources that still remain to be fully exploited for understanding ideologies in today's social world. It will benefit from the conceptual complexity of the systems-theoretical approach that helps to understand the diverse ways in which ideologies are implemented in the highly differentiated and reflective communicative processes of modern society, as it allows us to identify heterogeneous, and at times subtle, communicative acts and forms as ideological phenomena. Of course, an appropriate methodology for this still needs to be developed. It must combine objective hermeneutics, functional analysis, and a constructivist social epistemology for an analysis of functionally constituted and socially constructed operationalizations of meaning—a task for which Luhmann's analysis of love (Luhmann, 1986) may already serve as a model. For such an analysis, however, necessary criteria should be clarified now. They are found in the attribution of instructive, expressive, and/or reflexive functions to structures and operations in connection with other, underlying structures and operations with functions of the same kind. In most cases, ideological labels such as ‘liberalism' or ‘socialism' have already become established for compositions like these. Yet new ideologies may also be identified and observed in places where they are not immediately apparent. Given that, there is obviously still a lot to observe with systems theory.

## Footnotes

1 A more detailed discussion of these foundations and their potential—particularly their critical aspects—can be found in Klement (2026a).

2 However, functional equivalency does not preclude the possibility that structures coexist and interact. Consequently, ideologies should not be pitted against law, science, or politics per se, but also viewed in terms of functional complementarity, as we will see below.

3 In his original considerations, Luhmann describes the aspects of integration and coordination as the problem of organizational “Willensvereinheitlichung” (unification of wills) (Luhmann, ZK I, 1952–1960, 17-1)—a hint at the roots of his theoretical work in political science and organizational studies, which correspond with his biographical background. Hence, it should be evident that a theory of ideology based on Luhmann's work differs from other theories of ideology due to its conception of ideology-related problems. While Marxist or post-structuralist theories often link problems to (economically driven) needs and interests in domination and exploitation, the systems-theoretical approach conceptualizes the notion of a problem more abstractly, thereby opening it up to a greater variety of social problems—such as species reproduction—without reducing all of them to problems of domination and exploitation.

4 This does not exclude a ‘false consciousness' but rather implies it since the belief in a consideration of personal interests and needs serves to foster ideological consent.

5 This is particularly evident in the fact that ideologies do not have to be consistently ordered or communicated, as Luhmann notes (for a comprehensive discussion, see Klement, 2026a, p. 254–286).

6 Because of this lack of clear demarcation, ideologies can exert influence within social systems such as organizations, politics, the economy, science, and education. A systems-theoretical analysis of these multisystemic implementations has yet to be conducted.

7 All examples given here are for illustrative purposes only. Detailed studies on the role of ideologies during the COVID-19 pandemic from a systems-theoretical perspective have yet to be conducted. Such studies would need to examine, for instance, what consequences the prioritization, justification, and implementation of restrictive health protection measures—as opposed to common practices of freedom—have had on social systems such as health, politics, the economy, or education (see, for an overview of consequences, e.g., Nassimov et al., 2024; for early systems-theoretical approaches, Henkel, 2020; Stichweh, 2020). More specifically, one might ask how liberalism, in elevating the value of health, has reinforced political problems of legitimation and polarization while simultaneously responding to these problems, or how it intensified a contradiction between mental and physical health as a problem for the healthcare system.

8 In Luhmann's terminology, however, we would need to examine more closely the semantics and programming of guiding values, as expressed, for example, in the distinction between positive and negative freedom. This allows for a more nuanced understanding of the overlap often observed among various ideologies regarding highly abstract values, as it considers the ‘correct' usage of these values.

9 This does not imply that the ideological orientation determines all organizational actions. The usual gap between ‘talk and action' may persist, since what matters is only the reference to functionally relatable communication, even if it is merely written down and never spoken aloud or put into practice.

10 Similar theories regarding the multireferentiality of ideological communication are referred to in social-psychological ideology research as “multidimensionality” (Jost et al., 2009, p. 310–315) or in Marxist theories under the term “multi-accentuality” (Mumby, 1989, p. 297, with reference to Volosinov, 1973 and Hall, 1985). The systems-theoretical approach specifies multiple references as references to different systems, each with distinct functional conditions, communication media, information processing schemes, expectations, and problem contexts. In this way, it combines the analysis of ideologies and their contexts with an analysis of complex social logics. In doing so, conflicts between individuals, groups, and classes are not ignored but regarded as only one notable facet of systemic communication contexts among others.

11 In line with this, Luhmann understands the post-structuralist concept of discourse as a scientific paradigm for the external observation of self-observing communication systems, which, however, cannot observe themselves ad infinitum in their self-observation, so that suspicious omissions must be generated (Luhmann, 2000, p. 321). The observation of the selective construction of the discourse topic by the discourse itself is thus theoretically embedded in the analysis of ideological discourses.

12 In doing so, the systems-theoretical approach can even build upon the Marxist critique of the reification of ideological symbols, which seeks to obscure their nature as social constructs. It is no coincidence that Luhmann viewed the critique of reification as one of the lasting insights to be adopted from Marx (Luhmann, 1992a, p. 23–24). Unlike Marxism, however, a theory following Luhmann does not reduce ideologies to pejorative phenomena such as false consciousness but rather discusses reification from a functional perspective with a sense for (better) functional equivalents (Luhmann, 2005; Klement, 2026a, p. 200–253). As mentioned above, it does not reduce the understanding of ideological problems to domination, but also considers the problem of generalized social orientations to be a relevant issue, for which the reification of orientation symbols may be one possible option among others.

13 As mentioned above, a common interpretation of these problems includes, for example, interests and needs such as domination and exploitation. Ideological conflicts thus often appear as power struggles. The systems theory approach does not fundamentally contradict this view but rather expands it to a wider variety of conflicts between different problems and systems. Consequently, the ideological conflicts of modern society should also be interpreted as conflicts between different functional systems.
